# Neuropsychological function is improved among opioid dependent adults who adhere to opiate agonist treatment with buprenorphine-naloxone: a preliminary study

**DOI:** 10.1186/s13011-017-0133-2

**Published:** 2017-11-15

**Authors:** Travis M. Scott, Monica Rivera Mindt, Chinazo O. Cunningham, Franchesca Arias, Kelly Coulehan, Aprille Mangalonzo, Pat Olsen, Julia H. Arnsten

**Affiliations:** 1 Department of Psychology, Fordham University, Bronx, NY 10458 USA; 2 Department of Medicine, Albert Einstein College of Medicine and Montefiore Medical Center, Bronx, NY 10467 USA; 3 Department of Psychology, University of Florida, Gainesville, FL 32611 USA

**Keywords:** Opioid agonist treatment, Depression, Adherence, Neuropsychological change, Buprenorphine/Naloxone, Opioid use disorder

## Abstract

**Background:**

Among persons with opioid use disorder (OUD), neuropsychological dysfunction is associated with depression, and better neuropsychological function is associated with opioid abstinence. However, it is unknown whether depressive symptomatology or adherence to opiate agonist treatment are associated with neuropsychological change over time.

**Methods:**

We recruited 20 buprenorphine/naloxone-treated adults with OUD (*M* Age = 45.2 years [*SD* = 8.1]; 25% female) to complete baseline and 6 month visits containing a neuropsychological test battery and self-reported measures of depressive symptomatology and medication adherence.

**Results:**

Depressive symptomatology was not significantly related to neuropsychological change (*p*’s > .05). Greater adherence to buprenorphine/naloxone was associated with improvements in learning, memory, and global functioning (*r*’s = .52–60; *p*’s < .05).

**Conclusions:**

Among OUD patients, greater adherence to buprenorphine/naloxone is associated with improved neuropsychological functioning over time. In contrast, depressive symptomatology is not associated with neuropsychological functioning over time. Supporting adherence to buprenorphine/naloxone may improve and/or preserve learning and memory functioning in individuals treated for OUD.

**Trial registration:**

NCT01108679. Registered 21 April 2010.

The most common treatment for opioid use disorder (OUD) is opioid agonist therapy (OAT) with methadone or buprenorphine/naloxone, but little is known about how treatment with buprenorphine/naloxone impacts neuropsychological functioning among persons with OUD. Several factors may affect changes in neuropsychological functioning over time in OUD patients receiving treatment with OAT, including depressive symptomatology and medication adherence Exploring these factors in OAT-treated OUD patients might clarify the longitudinal impact of buprenorphine/naloxone treatment among persons at high risk for neuropsychological dysfunction.

## OAT and neuropsychological functioning

In persons with current OUD, neuropsychological impairment is common [1], but data suggest that both methadone and buprenorphine/naloxone improve neuropsychological functioning over time in a variety of neuropsychological domains (i.e., verbal learning, visuospatial memory, processing speed, executive functioning) [2, 3]. Improvements in neuropsychological functioning have been identified as early as 2 months after OAT initiation [2]. Preliminary studies indicate that patients taking buprenorphine appear to have better neuropsychological functioning than methadone patients, with medium to large effect sizes in learning and memory, attention/working memory, and executive functioning [4, 5]. While the results of these studies suggest that buprenorphine/naloxone may improve neuropsychological functioning over time among OUD patients, studies have not examined whether greater medication adherence results in even greater changes in neuropsychological functioning.

## Depression and neuropsychological functioning in persons with OUD

Depression is more prevalent in individuals with OUD than in the general population [6], but little is known about associations between depressive symptomatology and neuropsychological functioning in persons with OUD. Among other substance use disorder populations (e.g., alcohol, cocaine), depressive symptomatology is negatively associated with executive functioning [7], but few studies have examined depression and neuropsychological functioning among persons with OUD specifically. One recent study of opioid-dependent persons found a negative relationship between depressive symptoms and reaction time [8], but more research is needed to understand whether depressive symptomatology attenuates neuropsychological improvement among OUD patients receiving buprenorphine/naloxone treatment.

## OAT adherence and neuropsychological functioning

OAT adherence is typically operationalized as retention in substance use treatment [9], but how OAT retention affects neuropsychological outcomes over time is not well understood. Studies that have collected neuropsychological data from OAT patients have not examined how medication adherence during treatment relates to changes in neuropsychological functioning [2, 3]. To date, no studies have examined associations between OAT medication-taking adherence and neuropsychological outcomes over time.

## Study aims

We conducted this preliminary study to examine the longitudinal impact of depressive symptomatology and medication adherence on neuropsychological function among OUD patients in treatment with buprenorphine/naloxone. Our overarching hypothesis was that improvements in neuropsychological functioning over time would occur in: (1) persons with lower depressive symptomatology, and (2) persons with greater medication adherence. We also hypothesized that greater adherence to buprenorphine/naloxone would be independently associated with improved neuropsychological functioning.

## Methods

### Participants

This preliminary analysis was part of a larger pilot study designed to inform a randomized clinical trial examining neuropsychological effects of methadone and buprenorphine. The pilot study enrolled only participants who were newly initiating buprenorphine/naloxone treatment, who were recruited through word of mouth, flyers, and health care provider referrals.

### Inclusion and exclusion criteria

Inclusion criteria were: 1) current OUD per medical assessment by a physician; 2) no buprenorphine use for 15 consecutive days prior to study enrollment; and 3) anticipated buprenorphine start date within 30 days of study enrollment. Exclusion criteria included: 1) diagnosis of severe psychiatric or medical conditions (e.g., schizophrenia, stroke, head injury with loss of consciousness >12 h, or seizure disorders); 2) age younger than 18 or older than 65; 3) less than 6 years of education; 4) unable to speak English; or 5) not completing all neuropsychological, psychiatric, and adherence measures at the baseline and six-month visits.

### Procedures

Prior to completion of any study evaluations, all participants provided signed informed consent and agreed to complete all neuropsychological, psychiatric, and adherence measures at both the baseline and follow-up visits. After study enrollment, participants underwent buprenorphine/naloxone induction at a community-based clinic which provides integrated substance use disorder and medical care and treatment. After induction, participants were maintained on 4–16 mg of buprenorphine/naloxone for the six-month duration of the study. Specifically, all participants were in regular medical care and were stable on their buprenorphine/naloxone regimens throughout the study period. Participants received standard-of-care treatment for opioid use disorder and other medical conditions at the same clinic.

Participants completed the measures detailed below at both the baseline and six-month visits. Each visit, including the 90-min neuropsychological test battery, was completed in a single session and took approximately 4 h. Participants were provided with both a snack break and a lunch break during the study visits, and additional breaks as needed. Baseline visits were completed within 14 days of treatment induction, and six-month follow-up visits occurred between 165 and 225 days after baseline. The study was approved by the Institutional Review Boards of Albert Einstein College of Medicine and Fordham University.

### Measures

#### Depressive symptomatology

At both the baseline and six-month visit, participants completed the Beck Depression Inventory (BDI)-II, which evaluates depressive symptoms over the prior 2 weeks and produces a summary score [10]. We calculated a mean of the two summary scores to estimate depressive symptomatology over the 6 month study period.

#### Buprenorphine-naloxone adherence

The Visual Analogue Scale (VAS) measured self-reported adherence over two different 4-week periods, once at the three-month mid-point and once at the six-month follow-up visit. At each time point, participants were asked to look at the visual scale and “select the percentage that represents how much of your buprenorphine you’ve taken in the past 4 weeks.” For each participant, we then calculated a mean adherence rate based on their two VAS responses to reflect adherence over the 6 month study period. The VAS is highly correlated with electronic adherence monitoring systems and is well-validated as a measure of antiretroviral adherence [11, 12].

#### Substance use

Substance use disorder (SUD) diagnoses were determined through computerized structured clinical interviews, the Composite International Diagnostic Interview (CIDI) Version 2.1. Substance use severity was assessed with the Addiction Severity Index (ASI). The CIDI provides diagnostic information based on Diagnostic and Statistical Manual of Mental Disorders (DSM)-IV criteria [13]. The ASI is a structured computerized interview that assesses seven primary domains including alcohol and drug use [14].

#### Neuropsychological functioning

At both the baseline and six-month visit, participants completed comprehensive neuropsychological evaluations, including well-validated measures in the following domains: verbal fluency, processing speed, attention/working memory, learning, memory, motor functioning, and executive functioning (see Table 1 for complete list of neuropsychological measures). Estimated premorbid IQ was assessed with the Wide Range Achievement Test-3 Reading Subtest (WRAT-3). Trained psychometrists administered/scored all batteries and followed standardized procedures. Alternate forms of the learning and memory measures were used for the two visits.

**Table 1:** Neuropsychological battery and normative data by seven major areas for computation of T-scores and sRCS

Neuropsychological Domain and Test	Normative Data Sources [22–27]
Verbal Fluency	
Controlled Oral Word Association Test (FAS)	Heaton, Miller, Taylor & Grant (2004)^a,b,c,d^
Semantic (Animal) Fluency	Heaton, Miller, Taylor & Grant (2004)^a,b,c,d^
Speed of Information Processing	
WAIS-III Digit Symbol	Heaton, Taylor & Manly (2003)^a,b,c,d^
WAIS-III Symbol Search	Heaton, Taylor & Manly (2003)^a,b,c,d^
Trail Making Test (Part A)	Heaton, Miller, Taylor & Grant (2004)^a,b,c,d^
Attention/Working Memory	
WAIS-III Letter Number Sequencing	Heaton, Taylor & Manly (2003)^a,b,c,d^
PASAT Total Correct	Diehr et al. (2003)^a,b,d^
Learning	
Hopkins Verbal Learning Test - Revised	Benedict, Schretlen, Groninger & Brandt (1998)^a^
Brief Visuospatial Memory Test - Revised	Benedict (1997)^a^
Memory	
Hopkins Verbal Learning Test - Revised	Benedict, Schretlen, Groninger & Brandt (1998)^a^
Brief Visuospatial Memory Test - Revised	Benedict (1997)^a^
Motor	
Grooved Pegboard Time	Heaton, Miller, Taylor & Grant (2004)^a,b,c,d^
Executive Functioning	
Wisconsin Card Sorting Task-64 Item	Kongs, Thompson, Iverson & Heaton (2000)^a,b^
Trail Making Test (Part B)	Heaton, Miller, Taylor & Grant (2004)^a,b,c,d^

*Notes*. Wechsler Adult Intelligence Scales (WAIS); Paced Auditory Serial Arithmetic Test (PASAT); Summary regression-based change scores (sRCS); Normative data corrects for the demographic characteristics indicated by superscript: ^a^Age; ^b^Education; ^c^Gender; ^d^Ethnicity

To assess longitudinal changes in neuropsychological function, summary regression-based change scores (sRCS) were calculated. The sRCS approach accounts for practice effects and utilizes an electronic normative database in which individual z-scores at different time points are computed by dividing the difference between predicted and obtained follow-up scaled scores by the error term of the regression model [15]. The resulting z-score reflects how well or poorly the participant performed at the follow-up time point relative to expectations made from his/her prior score and other variable-specific baseline predictors. The difference between the predicted retest performance and actual retest performance on each individual neuropsychological test is then converted into sRCS. For this analysis, sRCS of individual neuropsychological tests from each domain were averaged to create mean domain sRCS, and all individual test sRCS were averaged to create a mean global sRCS. A 90% confidence interval was used to determine significant global neuropsychological sRCS change, with the top 5% considered significant improvement and the bottom 5% considered significant decline.

### Statistical analyses

The independent variables were depressive symptomatology (mean BDI-II summary score over two time points) and adherence to buprenorphine/naloxone (mean adherence rate calculated from two VAS ratings), and the dependent variables were changes in global and domain-specific neuropsychological functioning (global and domain sRCS, with positive scores indicating improvement). We computed Pearson correlations to examine the associations of depressive symptomatology and adherence with global neuropsychological and domain-specific sRCS.

To assess for potential covariates, we examined associations between sRCS and demographic characteristics, Wide Range Achievement Test-3 (WRAT-3) Reading T-score, urine toxicology, current substance use disorder, days of substance use in past month, and HIV-status. The only significant findings were that gender was related to global and processing speed sRCS (all *p’s* < .05), and that WRAT-3 Reading T-score was correlated with global and attention/working memory sRCS (all *p’s* < .05). Therefore, for all analyses that included the global, processing speed, or attention/working memory sRCS, we computed partial correlations to adjust for gender and WRAT-3 Reading scores.

All analyses were conducted using the Statistical Package for the Social Sciences (SPSS) Version 22.0. Alpha level was set at .05.

## Results

### Participant characteristics

Table 2 summarizes participants’ demographic and clinical characteristics. Participants were mostly male (75%), middle-aged, and had less than a high school education. Their mean estimated premorbid IQ based on WRAT-3 Reading was in the low average range (*M* = 86.9, *SD* = 14.0)*.* Most participants reported mild depressive symptomatology, but nearly one-third (30%) reported moderate symptoms. Approximately 28% were HIV+, and mean buprenorphine/naloxone adherence during the six-month study period was 91.3% (SD = 11.8).

**Table 2:** Participant demographic and selected clinical characteristics (*N* = 20)

	*M*(*SD*) or % (*n*)	Range
Demographic Characteristics		
Female	25% (5)	
Race/Ethnicity		
Hispanic/Latina/o	55% (11)	
African American	25% (5)	
Other/Not listed	15% (3)	
Non-Hispanic white	5% (1)	
Age	45.2 (8.1)	32–61
Years of Education	11.7 (2.3)	7–18
WRAT-3 Reading Subtest Standard Score^a^	86.9 (14.0)	66–109
HIV-Seropositive	28% (5)	
Depressive Symptomatology		
BDI-II Total Score^b^	13.2 (9.2)	0–29.5
% Adherence^c^	91.4 (11.8)	64–100

Notes

^a^WRAT-3 Reading = Wide Range Achievement Test-3 Reading Subtest standard score from baseline visit

^b^BDI-II Total Score = mean Beck Depression Inventory-II Total Score from baseline and 6 month visit

^c^Adherence = % mean adherence from visual analogue scale at two points (midpoint and six-month follow up visit)

Table 3 summarizes participants’ current and past substance use. Sixteen participants had a current substance use disorder (SUD) according to the CIDI, but all 20 had a lifetime SUD diagnosis. At baseline, 14 participants (70%) had a urine toxicology test that revealed substance use (i.e., amphetamines, benzodiazepines, cocaine, opiates, methadone, and/or oxycodone), and at the six-month follow-up visit 13 participants (65%) had a positive urine toxicology result. Three participants had negative urine toxicology results at both time points. Participants self-reported using heroin for a mean of 16.4 years (*SD* = 12.8), using methadone for a mean of 3.9 years (*SD* = 5.1), and using other opiates (e.g., opioid analgesics) for a mean of 4.2 years (*SD* = 8.1). In the month prior to the baseline evaluation, heroin was the most frequently used substance, with mean usage of 8.6 days (*SD* = 10.2). In the month prior to the six-month follow-up visit, other opiates were the most frequently used substance, with mean usage of 1.8 days (*SD* = 5.8).

**Table 3:** Participant current and past substance use characteristics (*N* = 20)

	*M*(*SD*) or % (*n*)
CIDI Lifetime Substance Use Disorder	
Cocaine	70% (14)
Alcohol	60% (12)
Cannabis	50% (10)
Hallucinogens	15% (3)
Sedatives	15% (3)
Positive Urine Toxicology Result (Baseline visit)	
Opiates	35% (7)
Cocaine	35% (7)
Methadone	20% (4)
Benzodiazepines	10% (2)
Oxycodone	10% (2)
Positive Urine Toxicology Result (6 Month visit)	
Opiates	42% (8)
Cocaine	31% (6)
Methadone	15% (3)
Amphetamines	11% (2)
ASI Days of Substance Use in Prior Month (Baseline)	
Alcohol Intoxication	3.2 (6.8)
Heroin	8.6 (10.2)
Methadone	2.3 (5.8)
Other Opiates	2.6 (5.5)
Benzodiazepines	0.5 (1.6)
Cocaine	3.5 (6.9)
ASI Days of Substance Use in Prior Month (6 Month visit)	
Alcohol Intoxication	1.3 (1.9)
Heroin	1.6 (3.2)
Methadone	0.1 (0.2)
Other Opiates	1.8 (5.8)
Benzodiazepines	0.4 (1.6)
Cocaine	1.2 (3.4)

*Notes*. *CIDI* Composite International Diagnostic Interview, *ASI* Addiction Severity Index

Table 4 summarizes participants’ neuropsychological (NP) characteristics. Over the course of 6 months, 15% of participants showed global NP improvement, 5% had global NP decline, and 80% remained stable. The mean global sRCS for the sample was −.01 (*SD* = .46), indicating no significant overall NP change. Changes in specific NP domains ranged from a mean sRCS of −.22 (*SD* = .50) in learning to a mean sRCS of .41 (*SD* = .92) in executive functioning, indicating minimal change in domain-specific NP functioning.

**Table 4:** Participant neuropsychological (NP) characteristics at baseline and follow-up (*N* = 20)

	Time 1	Time 2	sRCS
	T-score *M*(*SD*)	T-score *M*(*SD*)	*M*(*SD*)
NP Domains			
Global	41.8 (6.4)	44.5 (6.2)	−.01 (.46)
Learning	36.2 (11.7)	37.8 (9.3)	−.22 (.50)
Memory	36.9 (12.2)	38.5 (11.4)	−.12 (.89)
Verbal Fluency	46.3 (9.0)	47.3 (12.0)	−.13 (.94)
Processing Speed	49.5 (8.6)	52.2 (8.8)	−.09 (.88)
Attention/Working Memory	44.3 (8.0)	46.9 (9.2)	. 04 (.98)
Motor	39.2 (9.9)	42.9 (9.0)	.09 (.68)
Executive Functioning	41.7 (6.7)	46.2 (8.8)	.41 (.92)

*Notes*. *sRCS* summary regression-based change scores

### Depression, adherence, and neuropsychological functioning

Depressive symptomatology (mean BDI-II score) was not significantly correlated with global NP change (sRCS) or NP change in any NP domains (all *r*’s = −.02 to −.41; all *p*’s > .05), nor was it significantly correlated with adherence (*r* = −.05; *p* = .84).The results of remaining correlational analyses revealed that greater adherence to buprenorphine/naloxone was associated with improved learning sRCS (*r* = .52, *p* = .019), memory sRCS (*r* = .59, *p* = .006), and global sRCS (*r* = .60, *p* = .008), and these effects were in the medium range [16].

## Discussion

We found that self-reported adherence to buprenorphine/naloxone treatment over time is high, and that greater adherence is associated with improvement in learning and memory neuropsychological functioning. Although we found that adherence is also significantly related to improved global neuropsychological functioning, this finding was likely driven by the significant positive associations of adherence with learning and memory. By measuring OAT adherence over time and as a continuous variable, we have identified the unique impact of greater adherence on the neuropsychological domains of learning and memory.

Our finding that greater medication adherence is associated with improved neuropsychological functioning (i.e., learning and memory) over the course of 6 months in OUD patients is consistent with previous research [17, 18]. For example, one review article concluded that neuropsychological impairment is related to involuntarily dropping out of substance abuse treatment, and that duration of abstinence is correlated with improvement in neuropsychological functioning [18]. Similar to ours, another study found that patients receiving substance use disorder treatment (i.e., in a chemical dependency treatment program or in residential or intensive day treatment) have the greatest neuropsychological improvement over 6 weeks in the area of memory [17]. In contrast to prior studies [2, 3, 5], our study did not find a significant relationship between adherence to buprenorphine/naloxone and improved attention/working memory, processing speed, or executive functioning. However, prior studies did not examine the relationship between adherence and other domains of neuropsychological functioning, which highlights the specificity of our finding that adherence to buprenorphine/naloxone was uniquely associated with learning and memory, but not with other neuropsychological domains. This represents a highly novel finding for the field that warrants further study. Our study also extends prior findings by defining treatment adherence beyond program attendance, and by examining both adherence to medication-taking and neuropsychological functioning over a significantly longer period. Additionally, other studies have not explored the impact of other important variables, such as depression, on neuropsychological functioning over time.

Unlike prior studies [7, 8, 19], we did not find significant associations between depressive symptomatology and neuropsychological change over time, despite nearly one-third of the sample reporting moderate depressive symptomatology. However, the prior cross-sectional study by Beatty et al. [7] was conducted within a sample of patients with alcohol and cocaine use disorders, not patients with OUD. Another prior study by Horner et al. [19] did not use objective tests of neuropsychological function, instead measuring neuropsychological functioning through self-evaluation in a broadly defined sample of substance use disorder patients. A previous study by Loeber et al. [8] reported a significant association between depression and neuropsychological functioning in OUD patients using objective neuropsychological tests, but this foreign study had methodological differences from ours (e.g., longitudinal design, different neuropsychological measures). In addition, a review article concluded that the association between depression and neuropsychological functioning in patients with substance use disorders is equivocal [18].

Our study has several important implications. First, it advances understanding of neuropsychological outcomes among persons with OUD by quantifying OAT adherence rather than measuring attendance in a treatment program [17]. We have thus identified a potential target (medication-taking) to intervene to improve neuropsychological outcomes in a population with high rates of cognitive impairment. Second, unlike most prior studies, our longitudinal design enhances the predictive validity of our findings. Third, we used well-validated measures of depressive symptomatology and neuropsychological function, addressing limitations with prior research (e.g., Horner et al. [19]). Fourth and finally, the inclusion of a U.S.-based, ethnically diverse sample permits generalizability to those most at risk for depressive symptomatology and SUD. Though prior research suggests that ethnically diverse individuals have higher rates of mild and moderate depression than non-Hispanic whites [20] and are overrepresented among those with OUD in low-income areas [21], there is little research on the impact of OAT and depressive symptomatology on neuropsychological functioning in this population.

Despite its strengths, our study has limitations. The racial/ethnic diversity of our sample is both a strength and a limitation, as it is unknown whether our findings are generalizable to a predominantly non-Hispanic white sample. While this study provided a comprehensive overview of participants’ current substance use, an additional limitation was that information about other prescribed medications (unrelated to OAT) was not available. Because we did not have a non-treated control group, our conclusions are limited regarding how study participants compare to OUD patients not receiving treatment with buprenorphine/naloxone. However, our analyses accounted for many demographic characteristics known to impact neuropsychological test performance. Because this is a preliminary study, these findings should inform future, larger-scale studies in which non-treated control groups could be added. Considering that eight correlational analyses were performed, the .05 alpha level used in the present study is a limitation. However, applying an alpha correction, such as Bonferroni, would have significantly reduced the power given the limited sample size. Finally, medication adherence was only assessed through self-report. Given that there is no ‘gold standard’ for adherence to buprenorphine-naloxone, future studies would benefit from multiple measures of adherence to better contextualize the current findings.

Future studies should replicate these findings with a larger and more diverse sample. Additionally, research should explore the relationship between buprenorphine/naloxone treatment and other specific areas of neuropsychological functioning (e.g., reaction time) that have been associated with depression in OUD patients (e.g., Loeber et al. [8]) Lastly, studies should continue to explore the relationship between medication-taking adherence and neuropsychological functioning over time, using different medications and/or different ways of measuring adherence. For example, future longitudinal studies might use objective measures of adherence to examine the relationship between OAT adherence and neuropsychological functioning, or might test interventions, such as directly observed therapy, to enhance adherence.

## Conclusions

In sum, we found that greater adherence to buprenorphine/naloxone treatment is associated with improved learning and memory over time among persons with OUD. This finding illustrates the importance of medication adherence in improving neuropsychological functioning within this population. Supporting OAT adherence may be a particularly important component of improving and/or preserving learning and memory functioning in individuals treated for OUD.
